# Implementing and Delivering Culturally Centred Pharmacy Services Tailored to Ethnically Minoritised Populations: A Qualitative Systematic Review and Meta‐Ethnography

**DOI:** 10.1111/hex.70165

**Published:** 2025-01-31

**Authors:** Caitlin Shaw, Ghalib Khan, Thorrun Govind, Anna Robinson‐Barella

**Affiliations:** ^1^ School of Pharmacy Newcastle University Newcastle upon Tyne UK; ^2^ Health and Inequality Research Champion, Patient and Public Involvement and Engagement, School of Pharmacy Newcastle University Newcastle upon Tyne UK; ^3^ Population Health Sciences Institute Newcastle University Newcastle upon Tyne UK

**Keywords:** Communication barrier, Cultural competency, Health inequities, Pharmaceutical services, Qualitative research, Racial and ethnic minorities

## Abstract

**Introduction:**

Health inequities disproportionately affect people from ethnic minority communities and require a comprehensive effort across healthcare disciplines to tackle them. Ethnically minoritised populations continue to be underserved, despite the growing awareness of the detrimental link between ethnicity and poorer health‐ and medication‐outcomes. Pharmacy has been recognised as an accessible and inclusive healthcare setting, with the ability to meet diverse patient needs. Yet, there still remain distinct gaps in knowledge of how to best design, implement and deliver culturally centred pharmacy services for members of ethnic minority communities.

**Methods:**

A systematic literature search was undertaken in November 2023, across four databases: MEDLINE, Embase, CINAHL and PsycINFO. Qualitative studies were included if they addressed barriers, enablers and interventions aimed at tackling medicines‐ and health service inequalities affecting people from ethnic minority communities. Study quality was assessed using the Joanna Briggs Institute critical appraisal checklist. Data were synthesised using a meta‐ethnographic approach, according to Noblit and Hare, forming a qualitative evidence synthesis to further understanding.

**Results:**

This meta‐ethnographic systematic review synthesised data from 13 international studies eligible for inclusion. Four overarching third‐order constructs (termed ‘themes’) were developed through reciprocal translation and focused on: (i) navigating pharmacy systems across the globe; (ii) understanding cultural needs and beliefs that may influence medicine use; (iii) strengthening relationships with pharmacists and other healthcare providers and (iv) addressing possible language and communication barriers.

**Conclusion:**

Pharmacists and policymakers should aim to raise awareness of pharmacy services, increase the provision of cultural competency training within the profession, build stronger relationships with minority communities, and facilitate access to interpretation services. A template of recommendations has been developed to further implement and deliver such services on an individual pharmacy‐, community‐ and profession‐basis. Future research should seek to utilise lived‐experience narratives and participatory co‐design methods to further explore ways to address wider healthcare accessibility inequalities for this minoritised population.

**Patient or Public Contribution:**

Public contributors and authors (inequity research champions, G.K. and T.G.) informed and shaped this project during study design and conceptualisation; they helped to ensure that the study was conducted, and the findings were reported, with sensitivity.

## 1 Introduction

Health inequities disproportionately affect people from ethnic minority groups and require a comprehensive effort across healthcare disciplines to tackle them [1, 2]. Evidence has demonstrated higher incidences of adverse drug events, medication dosing errors and healthcare‐associated infections amongst people from ethnic minority groups, when compared to their White counterparts [3, 4]. There has been a higher prevalence of chronic illnesses reported in certain ethnic minority groups, including hypertension and stroke in Black Caribbean and African populations, and heart disease in South Asian groups [5]. Furthermore, people from ethnic minority communities have experienced increased barriers to healthcare access, as well as less effective healthcare provision [6, 7, 8, 9]. The COVID‐19 pandemic was especially pertinent in highlighting ethnic health inequities, where those people living in areas of highest deprivation were disproportionally affected [10, 11, 12, 13].

Tackling health inequity [14, 15, 16, 17], delivering culturally competent healthcare services [18, 19, 20, 21, 22], and tackling medicines‐related inequality [23, 24, 25] has received much attention in recent international literature across a number of healthcare settings and professional groups. One such setting that has been recognised as a place of accessible, inclusive and equitable healthcare is pharmacy [23, 26, 27, 28]. Pharmacists are often considered the most accessible healthcare professionals [29, 30], providing individualised health services without the need for appointments [31]. The accessibility of community pharmacies, in particular, has extended beyond their physical proximity and placement within local areas [32]; evidence has attributed their accessibility more broadly, such as delivering inclusive methods of communication and the ability to meet diverse patient needs through services including medication management, preventative care, and health education [25, 33, 34, 35, 36]. Yet, there still remain distinct gaps in knowledge of how to best design, implement and deliver culturally centred pharmacy services for members of ethnic minority communities [37, 38, 39, 40].

Ethnically minoritised populations continue to be underserved, despite the growing awareness of the detrimental link between ethnicity and poorer health and medication outcomes [41, 42]. To begin taking steps to close this inequality gap, and enhance healthcare services for those from ethnic minority communities, it is critical to better understand, recognise and address the barriers these individuals face. Unlike other methods of qualitative synthesis, meta‐ethnography allows the reinterpretation of themes from the primary studies, whilst developing and generating higher‐order themes and theories [43]. This meta‐ethnographic review aimed to synthesise existing qualitative research to gain a deeper understanding of the barriers, enablers and examples of interventions seeking to provide culturally centred pharmacy services tailored to individuals from ethnically minoritised communities.

## 2 Method

This meta‐ethnography and systematic review has been reported in accordance with the ‘Preferred Reporting Items for Systematic Reviews and Meta‐Analyses (PRISMA)’ guidelines (see Appendix A in the Supporting Information) [44] and has been written in acknowledgement of the eMERGe reporting guidance for meta‐ethnography [43].

### 2.1 Eligibility Criteria

This meta‐ethnographic systematic review focused on culturally‐centred, pharmacy‐delivered healthcare services that were designed for, and tailored towards, people from ethnic minority communities (see Table 1). Therefore, only studies that focused on the experiences of people from an ethnic minority community, when accessing healthcare delivered by a pharmacist or member of the pharmacy team, were eligible for inclusion. In the context of this systematic review, ethnic minority communities were defined according to the global National Institute of Health (NIH) definition, mirroring recent work [23, 28, 46, 47].

**Table 1:** The *P*roblem, *I*nterest and *Co*ntext (PICo) framework used for this study [45].

Problem	The tailoring of culturally centred, inclusive, pharmacy services
Interest	The target population for the study was people from ethnic minority communities. While there are many definitions of ‘ethnic minority’, the focus population for this review was those who fit under the definition as given by National Institute of Health (NIH) [46, 47]; namely, ‘The minority racial and ethnic groups defined by the United States Office of Management and Budget (OMB) are: American Indian or Alaska Native, Asian, Black or African American, and Native Hawaiian or Pacific Islander. The ethnicity used is Latino or Hispanic. Although these five categories are minimally required, the mixed or multiple race category should be considered in analyses and reporting, when available. Self‐identification is the preferred means of obtaining race and ethnic identity’.
Context	Any medicines‐ or health‐centred service that is based within pharmacy settings, delivered by a pharmacist or a member of the pharmacy team.

Other inclusion criteria comprised: (i) studies conducted using qualitative methodology, (ii) with qualitative (or mixed‐method) data that explored the development or delivery of culturally centred pharmacy services, (iii) that included the perspectives of people from ethnic minority populations in the place/country that the study was conducted. There were no restrictions placed on the gender, sex, ethnicity or immigration status of participants in the studies. Study exclusion criteria comprised: (i) studies relating to experiences of a group that are not an ethnic minority population in the country where the study was conducted; (ii) studies relating to the development of the pharmacy student curriculum; (iii) studies that focused on other healthcare disciplines rather than pharmacy; (iv) any systematic, scoping or literature reviews, conference abstracts, theses and clinical trials; as well as (v) articles that did not include any qualitative data. No restrictions were placed on the search such as year of publication and language of writing, as the researchers wanted to elicit all perspectives of ethnic minority communities across the years and across the globe, given the relatively unexplored topic.

### 2.2 Search Strategy, Information Sources and Study Selection

A systematic literature search was performed on 23 November and re‐ran on 29 December 2023 across four databases: Medline, EMBASE, CINAHL and PsycINFO. Medical subject headings (MeSH terms) were used to create the search, with Boolean operators (AND/OR) and truncation applied to refine search hits; the search strategy was developed by two members of the research team, CS and AR‐B, alongside a medical librarian. The search involved a combination of terms and synonyms relating to ‘cultural competency’, ‘pharmacy’, ‘ethnic minority’ and ‘qualitative research’; the full search strategy can be found in the Supporting Information: Tables 2–5 of Appendix B. A search of the grey literature, using Google Scholar, and forwards‐backwards reference searching was performed (independently by C.S. and reviewed by A.R.‐B.). There were no restrictions or limits applied.

All search records were imported into the database EndNote (Version X9) for ease of management; this included manually handling search results, removing duplicates and performing screening. The title and abstracts of all papers obtained were reviewed independently by two authors (C.S. and A.R.‐B.). Full texts were retrieved for articles that met the inclusion criteria for further evaluation, and for those that could not be rejected without certainty. Full text articles were screened independently by two authors (C.S. and A.R.‐B.); a third author (T.G.) was available to resolve disagreements by discussion if they arose, however they did not. The PRISMA flowchart was used to organise the studies, including the exclusion of papers that did not meet the inclusion criteria.

### 2.3 Reading, Data Extraction and Quality Appraisal

Two authors (C.S. and A.R.‐B.) closely read and re‐read the included studies to ensure close familiarity with the work. Data extraction was performed independently by C.S., and reviewed by A.R.‐B., using a customised data extraction tool informed by previous research [19]. Extracted data included study location, aim and methodology/methods of data collection; participant characteristics; and study findings/original author's interpretation of the data. Quality appraisal was conducted by researcher C.S., and reviewed by A.R.‐B., using the Joanna Briggs Institute (J.B.I.) critical appraisal checklist for qualitative research [48]; no studies were excluded on the grounds of quality.

### 2.4 Researcher Reflexivity and Positionality Statement

When conducting research on health inequity, and its wider connection to ethnicity, it is important to acknowledge the positionality and reflexivity of the research team. Author C.S. is an undergraduate pharmacy student and A.R.‐B. is a pharmacist and researcher with a specialist interest in medicines inequity and working with minoritised groups facing marginalisation; both actively reflected on their own positionality during the review process, both being White British female researchers and acting as allies in tackling ethnicity‐related inequalities within pharmacy practice, and wider health care settings, for members of ethnic minority communities. The team was made up of two inequity research champions as active co‐researchers (G.K. and T.G., both members of ethnic minority communities and who champion to tackle inequalities) who ensured the systematic review was conducted, data analysed and results reported, in a culturally sensitive manner.

### 2.5 Analysis and Interpretive Synthesis

A meta‐ethnographic approach, determined by Noblit and Hare, was utilised for this review [49]. As is typical of meta‐ethnography, an interpretivist paradigm and framework informed the approach to synthesising qualitative data [50]; in doing so, this approach appreciates the primary data within each study to form conceptual ideas, and so considers individual experiences, allowing researchers to centre the views of those at the focus of the study [43]. Meta‐ethnography, as a form of qualitative evidence synthesis, is now widely used in health research and uses processes of reciprocal translation to move beyond the initial reported outcomes from the primary studies and provide a more comprehensive understanding of a subject [51]. The method follows 7 steps: getting started; deciding what is relevant to the initial interest; reading the studies; determining how they are related; translating the studies into each other; synthesising the translations; and expressing the synthesis, although these steps may overlap and run parallel to one another [43, 50, 52].​

Quotations (termed ‘first‐order constructs’ taken from the primary studies) and the original author's interpretations (‘second‐order constructs’) were extracted from the studies by authors C.S. and A.R.‐B., working collaboratively] [49]. They were compared to determine how they were related and translated into one another (reciprocal translation); this involved chronological comparison of first and second‐order constructs one paper at a time to develop new interpretations [51]. This enabled the development of themes (‘third‐order constructs’) and subthemes as part of this interpretive synthesis. The third‐order constructs seek to ‘go beyond’ the original interpretations of the primary studies to derive a deeper analytical understanding around the topic of medicines – and healthcare – inequality for people from ethnic minority communities. For the purpose of this work, the third‐order constructs have been termed ‘overarching themes’ and ‘sub‐themes’.

## 3 Results

### 3.1 Search Results

**Figure 1:**
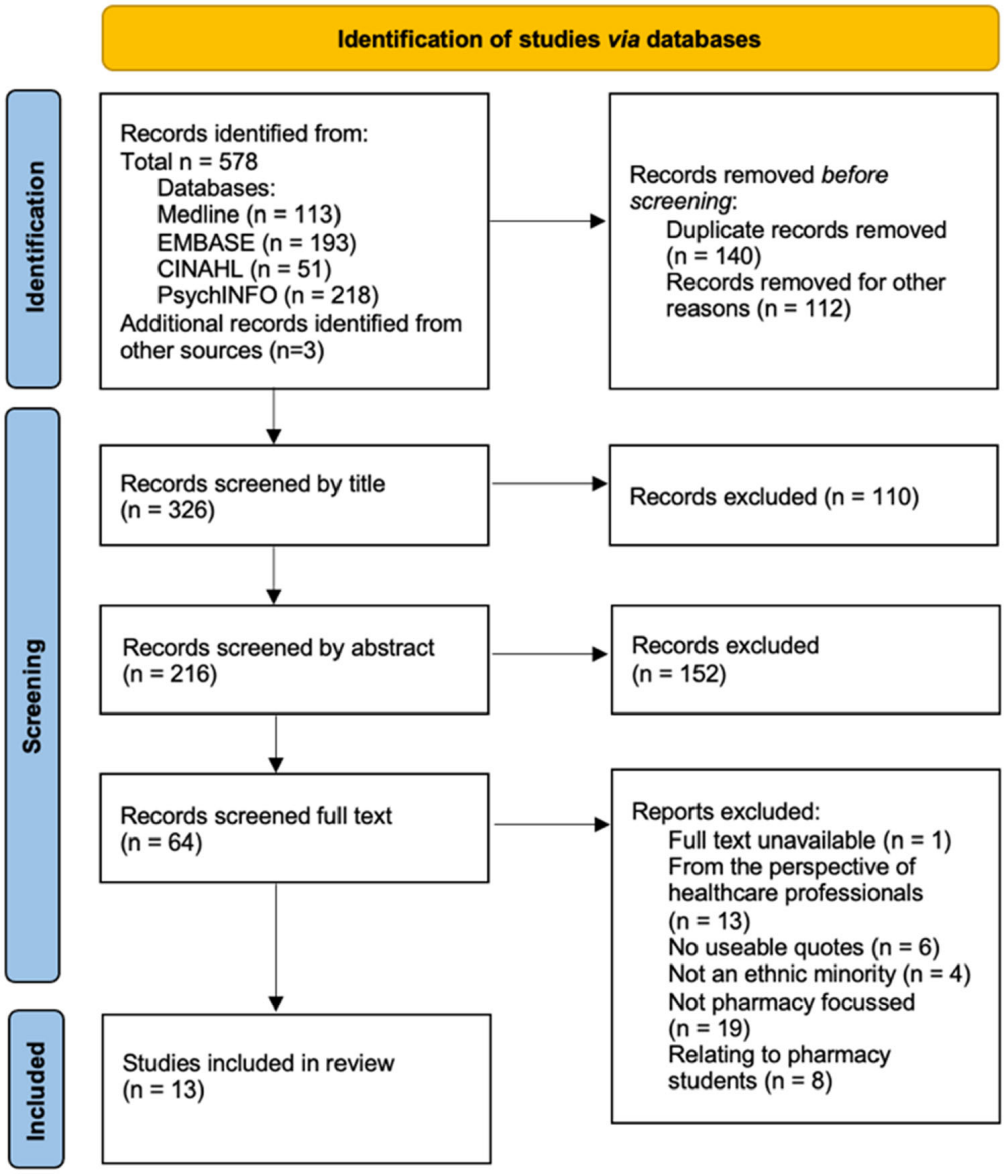
PRISMA flowchart for study selection and exclusion [44].

A combined result of 575 records were retrieved from database searches. Following the removal of duplicates and records in inappropriate study types and formats (including abstracts or theses), a total of 323 records underwent screening, of which 61 were selected to undergo full‐text review – of these, 51 studies were excluded, leaving 10 studies that were deemed to fit the inclusion criteria, alongside 3 studies identified from searches of the grey literature and reference‐checking. Figure 1 demonstrates the PRISMA flowchart for the study selection process, including reasons for study exclusion.

### 3.2 Study Characteristics

The final number of studies included was 13 studies. All included studies were published between 2012 and 2023, with the research being undertaken across 6 different countries: Australia (*n* = 5) [53, 54, 55, 56, 57], New Zealand (*n* = 3) [58, 59, 60], the United Kingdom (*n* = 2) [24, 28], Denmark (*n* = 1) [61], Canada (*n* = 1) [62], and the United States of America (*n* = 1) [63]. Sample sizes of the included studies ranged from 7 to 102 participants and included a range of different ethnic minority populations, including Indian, Chinese, Vietnamese, Somali, Congolese, Black, Arabic, Latina, Māori, and Aboriginal and Torres Strait Islander people, among others. The majority of studies focused on the experiences of people from ethnic minority groups when accessing pharmacy services, and how services were (or were not) tailored for them [24, 28, 53, 55, 58, 59, 60, 62, 63], however 2 studies related to experiences with a particular pharmacist‐led intervention aimed at educating ethnic minority populations [54, 61], and a further two studies related specifically to perspectives of the Australian healthcare service Home Medicines Review (a pharmacist‐facilitated service) [56, 57]. Table 2 outlines the key characteristics of all included studies.

**Table 2:** Study characteristics.

Study author and year	Location	Aim (verbatim from the original study)	Participant numbers and characteristics	Methodology, method of recruitment, data collection and analysis	Original authors overarching findings (mapped to second order constructs later)
Babar et al. [58]	Auckland, New Zealand	‘The aim of the study was to explore new migrants' knowledge, belief and attitudes of their experiences with regards to the access and use of medicines in NZ’.	Total 11 participants: all migrants	Qualitative research Snowball sampling Semi‐structured interviews Data analysis did not state a particular methodology	Financial barriers: paying doctor and pharmacist, lack of affordability of over‐the‐counter (OTC) medicines, sharing medicines Information transfer and knowledge of health systems Cultural and language barriers Perceptions of high quality of prescription medicines Non‐disclosure of traditional medicine use Variability of community pharmacy service provision, especially counselling
			7 of Indian ethnicity (4 female, 3 male) 4 of Chinese ethnicity (3 female, 1 male) Have lived in New Zealand for less than 5 years		
Bellamy et al. [53]	Queensland, Australia	‘The aim of this study was to explore the barriers to accessing medicines and pharmacy services among refugees in Brisbane, Queensland, Australia, from the perspectives of resettled African refugees’.	Total 16 participants: all resettled African refugees	Generic qualitative approach Purposive snowball sampling Focus groups Inductive thematic analysis	Health System Differences (regulation of medicine, getting medicines and identifying and treating the cause of symptoms) Navigating the Australian Health System (a confusing pharmacy system, navigating the pharmacy system and assimilating into the pharmacy health system) Communication Barriers [barriers to using the translating and interpreter system, lack of skilled professional communication and language skills (refugee)] Health‐Seeking Behaviour (preconceptions and preferences) and factors affecting engagement with Western medicine
			Somali Congolese Refugee community health leaders (RCHL) from South Sudan (2), Liberia, Eritrea, Uganda, and Burundi Resided in Australia for a range of 3–20 years All female participants except for one RCHL from Liberia		
Cantarero‐Arévalo et al. [61]	Copenhagen, Denmark	‘The aim is twofold: (1) to explore the perceptions, barriers and needs of Arabic‐speaking ethnic minorities regarding medicine use, and (2) to use an education program to enhance the knowledge and competencies of ethnic minorities in the appropriate use of medicines and prevention of MRPs’.	Total 30 participants: Arabic speaking ethnic minority	Cultural competence approach Purposive sampling Focus groups Data analysis did not state a particular methodology	Beliefs, perceptions and barriers related to appropriate medicine use: mistrust, language and alienation identified as barriers Participants needs concerning appropriate medicines use Issues specific to medicines and related topics Knowledge after the education programme
			27 female; 3 male 21–60 years 11 participants had lived in Denmark for more than 10 years; 3 had lived in Denmark for less than a year		
el Hait et al. [54]	New South Wales, Australia	‘The first aim of this study is to participants', who are Australian citizens of Arabic‐speaking origin, views about: taking medicines regularly, knowledge and feelings about diabetes, sources of medicines information, and special needs of being from an Arabic speaking background. The second aim is to explore participants' feedback about the use of the Diabetes Conversation Map as an educational tool in Arabic’.	Total 7 participants	Qualitative research Purposive sampling Focus groups Thematic analysis	Pertaining to adherence; knowledge and feelings about diabetes, special needs of being from an Arabic speaking background:
			Have Type 2 diabetes 54–73 years Arabic speaking Predominant country of birth was Lebanon; others from Syria and Egypt		
					Knowledge Relevance Communication with healthcare providers State of mine Mood Denial Needs Arabic speaking Healthcare providers Therapeutic needs Culturally relevant information on nutrition
					Pertaining to the specific intervention:
					Target audience Group education Language Difficulties with the Diabetes Conversion Map Goals
Gebre et al. [62]	Nova Scotia, Canada	‘We aimed to explore the experiences of Black Nova Scotians with community pharmacists’.	Total 16 participants	Qualitative research Purposive sampling Focus groups and interviews Thematic analysis	Difficulties navigating pharmacy as a Black person Lack of inclusivity and cultural competence in the pharmacy Transactional relationships
			Black Nova Scotians Majority 18–35 years Most resided in Nova Scotia since birth 15 female; 1 male All completed postsecondary education		
Hikaka et al. [59]	New Zealand	‘The aim of the current paper is to report patient acceptability of a pharmacist‐facilitated medicines review intervention for community‐dwelling Māori older adults’.	Total 17 participants	Mixed methods: quantitative and qualitative Purposive sampling Structured interviews (Likert scale + 2 open‐ended questions) General inductive approach	Medicines knowledge from a trusted professional Increased advocacy ‘By Māori, for Māori’ Increased confidence and control Financial and resource implications
			Māori 58–92 years 12 female; 5 male		
Hikaka et al. [60]	New Zealand	‘The aim of this study was to explore: the types of minor ailments Māori access care for; Māori experiences of access to medicines for minor ailments; Māori perceptions of the role pharmacists play in minor ailment care’.	Total 62 participants	Mixed methods: quantitative and qualitative Convenience and snowball sampling Wānanga (collaborative knowledge‐sharing group discussions) General inductive approach	Designing the right environment for minor ailment care Clinically and culturally safe care Moving from stigmatising to strengths‐based services The benefits of PMAS
			Māori 71% female, 29% male Age range 18–75+ (median age range 35–44 years)		
Knecht et al. [63]	California, USA	‘The objective of this study was to determine the nature and quality of the interactions between a cohort of Spanish‐speaking women with their community pharmacists and pharmacy staff in an Inland Empire community in Southern California’.	Total 24 participants	Qualitative, descriptive, cross‐sectional research Convenience sampling One‐on‐one interviews Inductive grounded theory approach	Language barriers Experiences with a pharmacist/pharmacy Obtaining and using medication Obtaining and understanding health information Suggestions for improvement
			Latina women No other personal demographic data was collected		
Mohammad et al. [55]	New South Wales, Australia	‘This study aimed to explore the needs of Australian CALD populations in regards to their medicines use, health information and utilization of pharmacist expertise in this context’.	Total 31 participants	Qualitative Purposive convenience sampling Semi‐structured interviews Grounded theory approach with constant comparison	Health information: usefulness of available health information; sources of health information; understanding medicine‐related instructions; differentiation between medicines Interactions with health care professionals: linguistic sanguinity and multilingual skill desirability; hierarchical social relationships Social networks Perceptions and beliefs influencing health‐related behaviour
			Language spoken at home: Arabic (2); Bengali (4); Czech (1); Filipino (1); Greek (1); Hindi/Punjabi (4); Indonesian (1); Malay (1); Maltese (1); Mandarin/Cantonese (10); Māori (1); Spanish (4) 20 female; 11 male Age range 25–65+ years		
Robinson et al. [24]	UK	‘When considering the value that medication reviews can offer in optimizing a person's medication, it is important to (i) understand what barriers may exist that impact the access of those from ethnic minority communities and to (ii) identify measures that may facilitate improved service accessibility for these groups’.	Total 20 participants	Qualitative Purposive sampling Semi‐structured interviews Reflexive thematic analysis	Building knowledge and understanding about medication reviews: lack of familiarity with medication review; raising awareness through community‐centred support The delivery of medication review services: addressing language and communication barriers; face‐to‐face connections and reassurance Appreciating the lived experience of patients: traditional, religious or cultural influences that affect medicine use; acknowledging cultural beliefs and recognizing potential stigma
			16 UK citizens 2 refugees 2 asylum seekers Average age 52 years 5 different ethnic groups represented: Asian or Asian British (7); Black, African Caribbean or Black British (3); White (3); mixed or multiple ethnic groups (3); other ethnic group (4)		
Robinson et al. [28]	UK	‘This study seeks to integrate the voices of those people from ethnic minority populations to gain better insight and create recommendations, on improving access to medicines advice from community pharmacies for people from ethnic minority communities’.	Total 12 participants	Qualitative Purposive sampling Codesign workshops consisting of 2–4 participants in each, separated by ethnicity. Reflexive thematic analysis	Delivering and providing culturally competent medicines advise: providing medicines advice tailored to diverse patient cohorts; appreciating medicine‐taking behaviours and cultural influences Building awareness of accessing medicines advice from community pharmacies: advertising and raising awareness in the community; geographical and financial barriers Enabling better discussions with patients from ethnic minority communities: verbal and nonverbal communication; building trust with communities
			8 UK citizens; 2 with residency visas; 2 asylum seekers Age range 33–72, average age 48 years 4 different ethnic groups represented: Asian or Asian British (4); White (identified as Jewish) (3); mixed or multiple ethnic groups (2); other ethnic group (3)		
Swain and Barclay [56]	Australia	“The goal of the present study was to explore Aboriginal and Torres Strait Islander perspectives of the HMR program and their suggestions for an ‘improved’ or more readily accessible model of service”.	Total 102 participants	Qualitative Purposive sampling Focus groups Data analysis did not state a particular methodology	Cultural considerations for Home Medicines Reviews with Aboriginal patients: ‘it works to be organised by the health service’; ‘it can't just be anyone’; ‘sometimes you don't want someone in your home’; ‘the health worker is the key’; group Home Medicines Reviews Adapting Home Medicines Reviews to Aboriginal patients' needs: explaining the process; referrals; medication specialists; written information
			Aboriginal and Torres Straight Islander people 75% female; 25% male 90% participants appeared to be over 40 years 23 participants had used the HMR programme; 79 participants had not		
White et al. [57]	New South Wales, Australia	‘This paper examines: (1) how aging HMR‐eligible Chinese and Vietnamese Australians who have never received a HMR manage their medicines; (2) to what extent they are aware of the existence of this free health service; and (3) how likely they might be to accept and receive a HMR in the future’.	Total 17 participants	Qualitative Purposive sampling Semi‐structured focus groups Data analysis did not state a particular methodology	Concerns about medicines Relationship with service providers Language barrier Awareness and attitudes towards HMR The GP as HMR barrier
			6 Chinese 11 Vietnamese HMR eligible 55–83 years		

Abbreviations: CALD = culturally and linguistically diverse; HMR = home medicine review; MRPs = medicine‐related problems; NZ = New Zealand; PMAS = pharmacist minor ailment services; RCHL = refugee community health leaders.

### 3.3 Quality Appraisal

Quality appraisal was carried out on all 13 studies included in this review (see Supporting Information: Appendix C, Table 6). Out of the 13 included studies, Bellamy et al. [53], Hikaka et al. [60] and Robinson et al. [28] were identified as being of the highest quality. The main weaknesses reported across the studies related to bias of the researcher: only four of the studies included a statement locating the researcher culturally or theoretically [59, 60, 61], and no studies included statement on the philosophical perspective of the research (question 1 of the JBI tool).

### 3.4 Findings: Reporting Outcomes, Synthesising Translations and Developing Themes and Subthemes

**Figure 2:**
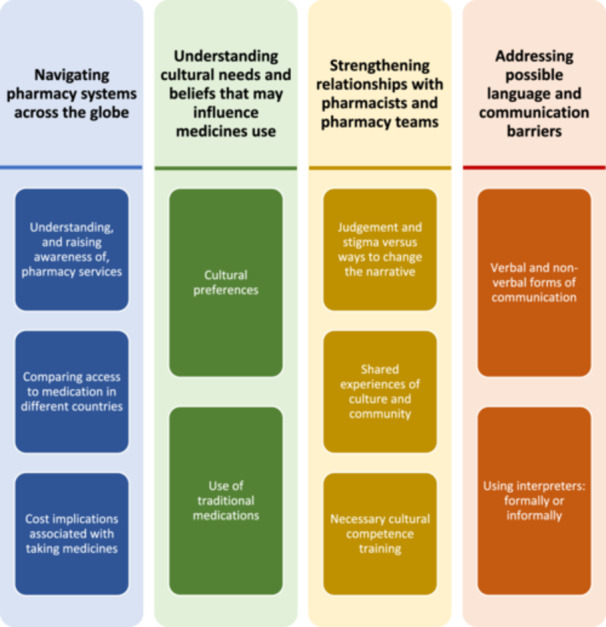
The developed themes and sub‐themes concerning the tailoring of culturally centred pharmacy services for those from ethnic minority communities.

Across the papers included in this meta‐ethnographic systematic review, there were 4 third‐order constructs developed (termed ‘themes’). All four themes appeared key when it came to delivering culturally centred pharmacy services tailored for individuals from ethnic minority communities (outlined in Figure 2). As is typical in a meta‐ethnography, each theme is presented in a table; these tables showcase examples of direct quotations (first‐order constructs) from study participants, the authors' interpretations from the original findings (second‐order constructs), and our interpretation as themes (third‐order constructs) and subthemes, moving beyond the findings of the original study. The four themes centred on: (i) navigating pharmacy systems across the globe (Table 3); (ii) understanding cultural needs and beliefs that may influence medicines use (Table 4); (iii) strengthening relationships with pharmacists and pharmacy teams (Table 5); and (iv) addressing possible language and communication barriers (Table 6). Each theme, and subsequent sub‐themes, are discussed in turn. From this, as an approach to begin tackling and addressing medicines‐related inequity, a template of key recommendations has been developed for stakeholders to support the implementation and delivery of culturally centred pharmacy services.

**Table 3:** Theme 1: Navigating pharmacy systems across the globe.

Overarching third‐order construct (termed ‘theme’) developed by the research team	Third‐order subtheme developed by the research team	Second‐order constructs (interpretations made by the original study authors)	First‐order constructs (direct quotations from the participants of the original studies).
Navigating pharmacy systems across the globe	Understanding, and raising awareness of, pharmacy services	Access to medicines in New Zealand [58]	‘*People don't really know anything about it [the medicine system in New Zealand] and we don't know where to go to get that help … it needs to be more open’.* (Respondent 1)
			‘*I see some families that are going to different pharmacies, husband going to one pharmacy, wife to another; they will never get a subsidy card’.* (Respondent 4)
		Other barriers to quality use of medicines [58]	‘*When you write two repeats before the date we do not understand what that means … this is difficult for us, we have paid for the whole amount why do we have to come back?’* (Respondent 5)
		Navigating the Australian Health System [53]	‘*If I come across a pharmacy, I show them and I tell them you know you can buy Panadol without a prescription and stuff but I don't tell them the actual process. I don't think nobody tells them the actual process’.* (Focus group 3)
		Needs concerning appropriate medicines use [61]	‘*There are no regulatory systems in our countries, so we need information about medicines’.* (Focus group 7)
		Selected medicine‐related topics [61]	‘*I have never heard about it [the reimbursement system]’.* (Focus group 2)
		On beliefs, perceptions and barriers [61]	‘*We are coming from a different country, culture and system, and we have to adjust to a new society. Everything is different’.* (Focus group 8)
		Health information: Sources of health information [55]	‘*I actually don't know what sort of health information I can get. So I don't know what to ask’.* (Participant 24)
		Building knowledge and understanding about medication reviews: Lack of familiarity with medication review [24]	‘*I got this feeling a lot of people doesn't really know what service the GP practice and the pharmacy do offer for medicines… He or she maybe doesn't know there is such a thing as a medicine review’.* (Participant 4)
			‘*When you come from India, you don't know that you need the (medicine) review. I just get medicines in India… no questions if they working… then the lady pharmacist here, she tells me I need one and I think, why?… But in England, it is different with having the review, then you learn to know much more and learn how such‐and‐such medicine works… you can check these things every year time, which I like better and I understand much more of the medicines now’.* (Participant 3)
			‘*(Healthcare professionals) maybe not know that we don't have this in our own country before… they maybe explain it better for us… then we can know to understand (the medicines review) is existing and why it is good for us patients to have it’.* (Participant 3)
			‘*When someone is at their most vulnerable, they need to know where to go for these things… making sure (the medications) are prescribed, making sure they are safe – that's all important’.* (Participant 19)
		Building knowledge and understanding about medication reviews: Raising awareness through community‐centred support [24]	‘*[My Rabbi could] indicate to the Jewish community, (medication reviews) is something we could be having to look after ourselves in a specific medicines‐way… (whilst) adhering to the principles of our religion’.* (Participant 11)
			‘*Community gatherings, not necessarily in a place of worship’* (Participant 8)
			‘*[Diverse geographical areas may hold diversity events that] could be useful because you'd have people from different groups there… it's about all‐community information for minorities’.* (Participant 19)
			‘*[Flyers in] the mosques and temples’*. (Participant 8)
		Building awareness of accessing medicines advice from community pharmacies: Advertising and raising awareness in the community [28]	‘*When I coming here from India, no such thing existed that I knew of in my home country… (but) we learn about asking the pharmacist when we came here to England’.* (Participant 7)
			‘*Improve the people's knowing about (this), especially when they first come to this island… they maybe don't know these things… it is important to help them know and learn because it is for their benefit’.* (Participant 9)
			‘*[Community‐wide advertising could help] ‘get the message out to the new arrivals… and also for people who maybe living here long time in UK but maybe not sure of’ the purpose, rationale and benefits of seeking medicines advice’.* (Participant 9)
			‘*Advertising the medicines review in the local shops, like my local Halal food store, where you'd get the footfall from the people who might benefit the most’.* (Participant 5)
			‘*The local Asian radio station even, they might spread the word about (medicine) reviews if you were the local (pharmacy)’.* (Participant 6)
			‘*Put posters up in the Mosque, they'll see them regularly… read them and probably talk about them with other people attending’.* (Participant 6)
			‘*Hearing about it from people they trust… like the Imam, or equivalent if they aren't Muslim, that's probably going to be someone in their community they listen to and respect’.* (Participant 5)
			‘“*Why do I have to go through my medical history with you?” but they might not understand the purpose of what it is for’.* (Participant 5)
		Geographical and financial barriers [28]	‘*Awareness on where (the community pharmacy) is, how to get there, which bus to use, those types of things’*. (Participant 1)
			‘*Even negotiating which bus to take to get there, which certainly isn't easy if you're new to the country, don't speak the language, and only receiving minimal (monetary) benefits’.* (Participant 6)
		Adapting Home Medicines Reviews to Aboriginal patients' needs [56]	‘*No one knows that it is available’.*
		Concerns about medicines [57]	‘*We got nobody to seek for advice and we don't know where to go’.*
	**Comparing access to medication in different countries**	Access to medicines in New Zealand [58]	‘*All I knew was that there was a health system here … I thought it would be the same as the medicine system in India; that you could just buy all medicines including antibiotics over the counter’.* (Respondent 1)
		Comparison of systems [58]	‘*You can just buy medicines over the counter [in India], you can buy antibiotics, analgesics anything just tell the pharmacist and they will get for you’.* (Respondent 1)
			‘*We had to get prescriptions, even if I knew what I wanted, you can't go directly to the pharmacy and get it’.* (Respondent 7)
			‘*In India there are so many options we can try you know what ever we want, doctors are not restricted like here. … Here if we do something different and it goes wrong we end up in hospital so we have to follow their treatment’.* (Respondent 3)
		Problems and challenges faced with medicines access: Lack of supply of traditional medicines [58]	‘*Basically no Chinese remedies are available and they had to bring their own remedies from China’.* (Respondent 8)
		Perceptions regarding medicines use: Use of traditional medicines versus modern medicines [58]	‘*You won't find all the preparations you find in India in New Zealand; there are a lot more preparations in India’.* (Respondent 5)
		Health system differences [53]	‘*Easier to get medicines in our country [in Africa]’* (Focus group 1)
			‘*Even some of the strong medicines here, like antibiotics, we don't call it strong back home because you buy it just from anyone at a shop without any script’.* (Focus group 4)
			‘*Back home when you get sick you don't have to go to the doctor, you can go to the pharmacy and you can explain to the pharmacy I have a headache, I have a tummy ache and they give you medicine’.* (Focus group 1)
		Obtaining and using medication [63]	‘*When we go to TJ [Tijuana] my mom gets these meds called XL3 for fever or cold or something like that and antibiotics’.* (Participant 8)
			‘*We used to go get medicine but now we have health insurance. We went to the doctor and bought medicine there [TJ] when we didn't have insurance; downtown TJ every corner there's a pharmacy’.* (Participant 24)
	**Cost implications associated with taking medicines Pharmacies as**	Comparison of systems [58]	‘*I see some people they say, ‘We cannot afford these’ and so they do not have them [medicines]’.* (Respondent 1)
		Perceptions regarding affordability [58]	‘*I can't go and buy that [OTC cold and flu medicine] because it is too expensive’.* (Respondent 10)
			‘*When we have to buy medicine even Panadol™ for about six dollars it is too much’.* (Respondent 1)
			‘*It is very hard to do everything with one income; you won't be able to afford all the medicines. … If it's a little bit [small cost] then we can manage, but before it was subsidised it was really hard’.* (Respondent 3)
			‘*In the initial phase when you're not a NZ resident, like on work permits still or just a visitor; at that time the doctors charge you more and you do not get subsidy on medicines at that time. … It's really not affordable at that time’*. (Respondent 1)
		Selected medicine‐related topics [61]	‘*I feel insecure about choosing the cheapest. I do not have enough money to choose the good quality product’.* (Focus group 1)

Abbreviation: OTC = over‐the‐counter.

**Table 4:** Theme 2: Understanding cultural needs and beliefs influencing medicine use.

Overarching third‐order construct (termed ‘theme’) developed by the research team	Third‐order subtheme developed by the research team	Second‐order constructs (interpretations made by the original study authors)	First‐order construct (quotations taken directly from participants of the original studies)
Understanding cultural needs and beliefs that may influence medicine use	Appreciating cultural and religious preferences regarding medicines	Perceptions regarding medicines use: Barriers to optimal medicines use [58]	‘*The medicine does the same thing but it is alcohol‐based … no one told us before … I would prefer to have all the options and then be able to decide’.* (Respondent 3)
		Language and cultural barriers [58]	‘*In India woman like to speak to female pharmacists and doctors … I am not comfortable talking to a male’*. (Respondent 4)
		Perceptions and beliefs influencing health‐related behaviour [55]	‘*In my country they always said if there is a bleeding cut, I shouldn't put any water but over here they say before I put any medicine or bandage I should wash the area with clean water … it's culturally different, totally opposite’*. (Participant 23)
			‘*Well I think the Chinese community has very different kinds of habits … For example when we take medicine we use lukewarm water but when I was hospitalized the nurses were all Westerners and gave me medication with tap water; I found it really hard to get used to it’*. (Participant 26)
		Appreciating the lived experience of patients: Traditional, religious or cultural influences that affect medicine use [24]	‘*The doctor focus only on the symptoms and the suitable medicine for helping me, but no one focuses on the medicine, if it is related to certain foods, then this can be a big problem for Muslim culture and Muslim religion… we might not take it, we might not want to discuss further with them… that is our beliefs’*. (Participant 16, via an interpreter)
			‘*Not translated on the labels on the medicine or mentioning by the doctor or pharmacist that medicines like this have the alcohol content… I am not allowed to take any alcohol at all as a Muslim and this is very, very important to me’*. (Participant 16, via an interpreter)
			‘*Sometimes (healthcare professionals) don't understand – I told them I'm a Sikh and I'm an Indian background, so he knew I could have the certain medicine that maybe the Muslims can't have with it being not Halal ingredients, but he didn't understand it… to me that is a big, big difference… this is why it is important for us to feel that they (healthcare professionals) respect and know our cultures and our backgrounds’*. (Participant 1)
		Appreciating the lived experience of patients: Acknowledging cultural beliefs and recognising potential stigma [24]	‘*Listening, empathy… learn about the person, be mindful of (their past) … accepting more that there could be something in their religion why they do something… like Ramadan and fasting and not taking tablets sometime’*. (Participant 18)
			‘*Any sort of mental health issuesas Jewish people are very, very closed about (mental health conditions)… when they come to marry, some people will be concerned’*. (Participant 11)
		Delivering and providing culturally competent medicines advice: Providing medicines advice tailored to diverse patient cohorts [28]	‘*Them not caring about me as a person… to me, these things need considered because it affects me as a person’*. (Participant 2)
			‘*My own preferences… these should be the things pharmacists and doctors consider for us (Arabic community) but maybe they don't know them, maybe they aren't taught them, but there's a simple way of asking me to find this stuff out’*. (Participant 2)
			‘*They didn't ask me, for example, if I have concerns with blood transfusions or if I have any beliefs related to the medicines that I would take’*. (Participant 2)
		Delivering and providing culturally competent medicines advice: Appreciating medicine‐taking behaviours and cultural influences [28]	‘*It could be like gelatine form, where the Indians are not supposed to take any material from cows or beef, it's a religious barrier for us’*. (Participant 7)
			‘*Does the medicine contain pork, does it have a gelatine capsule and does it contain alcohol? There's all of these different barriers, culturally and religiously, to make sure that people are looked after in a way that they are wanting to follow’*. (Participant 5)
			‘*Impact of times in religious calendars that medicines services should be aware of, like Ramadan and times of fasting’*. (Participant 4)
	Use of traditional medications	Perceptions regarding medicines use: Use of traditional medicines versus modern medicines [58]	‘*We know what is best. We know it doesn't have side‐effects [talking about natural/traditional remedies] … It doesn't harm anything so we just do it’*. (Respondent 3)
		Obtaining and using medication [63]	‘*I believe more natural products are better and should only use medicine for a serious illness or condition’*. (Participant 14)
			‘*The grocery store has a stand with medicine from Mexico, jarabe (syrup) for coughing, ointments containing zinc, and Pomada de la Campana. Those are found in Mexican grocery stores’*. (Participant 16)
			‘*I use chamomile tea, aloe vera and mint regularly. I have my herbs in my garden and my mom brings me dry herbs’*. (Participant 13)
		Appreciating the lived experience of patients: Traditional, religious or cultural influences that affect medicine use [24]	‘*It is the ancestor's beliefs; this is very important in the culture of my family… the peoples probably prefer to take something herbal or of the Chinese traditional medicines because they are familiar with it’*. (Participant 4)
			‘*First try the traditional remedies… only take the medicine if think traditional won't work’*. (Participant 7, via an interpreter)
			‘*The way I feel about asking their opinion with the medicines… if I know they have an idea of my culture then it's a better thing for me… no judgement if I take the herbals’*. (Participant 4)

**Table 5:** Theme 3: Strengthening relationships with pharmacists and other healthcare providers.

Overarching third‐order construct (termed ‘theme’) developed by the research team	Third‐order subtheme developed by the research team	Second‐order constructs (interpretations made by the original study authors)	First‐order construct (quotations taken directly from participants of the original studies)
Strengthening relationships with pharmacists and other healthcare providers	Judgement and stigma, versus ways to change the narrative	On beliefs, perceptions and barriers [61]	‘*We have a huge problem. Danish doctors ignore our needs. They never prescribe medicine for us, even when we are very sick’*. (Focus group 1)
			‘*When I go home, I don't take the prescribed medication, because I think that he [the Danish doctor] just wants to get rid of me. So, when I visit Morocco, I consult a doctor’*. (Focus group 6)
		Microaggressions and stereotyping [62]	‘*They definitely have a preconceived notion that people of colour don't have enough or [aren't] in the situation to pay for it [medication]’*.
		If I was White, it wouldn't be this complicated [62]	‘*I do understand if I was White, it probably wouldn't be this complicated. It would just go smoother, so I think it is very based on our skin colour’*.
		Language barriers [63]	‘*Some speak Spanish but they don't want to speak Spanish to me’*. (Participant 3)
			‘*I don't use them. I feel they don't want to talk to me’*. (Participant 2)
		Experiences with a pharmacist or pharmacy staff [63]	‘*The pharmacist gets angry when I ask if they can speak Spanish; it makes me feel bad’*. (Participant 5)
			‘*Sometimes when they do not speak Spanish, they make faces’*. (Participant 13)
		Acknowledging cultural beliefs and recognising potential stigma [24]	‘*(Appreciating) the possible past‐experiences that someone might have, because that isn't something they'll get over quickly. It's something that will be internalised and potentially affect them actually coming to (review their medications) if they know you'll be judgemental or not listen to them… show empathy… (take) time to listen*’. (Participant 5)
		Building trust with communities [28]	‘*Build that credibility amongst the communities, to show that expertise and open the dialogue’*. (Participant 6)
		Cultural considerations for home medicines reviews with aboriginal patients [56]	‘*Diffusing people's fear helps them to understand’*.
		Designing the right environment for minor ailment care [60]	‘*Knowing how to remove what is the perceived power balance between me as the Māori that goes in, versus that very flash looking Pākehā [NZ European] behind the bench that I think is looking down on me’*. (Participant 52)
			‘*At the moment, you walk in the [pharmacy] door, and you're assaulted by the stink of perfume … When you have a pharmacist who goes out into the community and meets you in a comfortable setting, not surrounded by those bright lights and shiny shelves, you're more likely to listen to what they're saying, [and the] advice on product, than someone that kinda looks like a retail salesperson’*. (Participant 14)
		Moving from stigmatising to strengths‐based services [60]	‘*I think one of the big stigmas for Māori and the lower economic whānau is the stigma of whakamā (shame). With those types of ailments that you spoke to … If we're gonna say that we're only going to fund those people that in the lower end of the pay scale, that's immediately gonna drag on a whakamā (shame) over them’*. (Participant 57)
		Building genuine relationships with Black patients [62]	‘*I was gonna say… sincere, like outreach. Not performative. If there was some substance behind reaching out to the Black community. Do they even know this is an issue? I think it's amazing you're doing this research but I'm also like you know, these pharmacies have been here for how long? How come this research has never been done? Like you've had Black customers. You know?’*
		Medicines knowledge from a trusted professional [59]	‘*[It was valuable to have] someone that knew what they were talking about. I was able to ask the things I wanted and felt comfortable asking her’*. (Participant 13)
	Shared experiences of culture and community	Acknowledging cultural beliefs and recognising potential stigma [24]	‘*People will not discuss it (mental health conditions) … especially if the pharmacist was a member of the community himself… that way it might become common knowledge and affect the family's reputation’*. (Participant 11)
			‘*I went to the GP for my medications and discovered that the doctor is Egyptian man. It was not easy … I didn't want him to have access to the list of depression medications I take… it is not something I wish my community (members) to know*’. (Participant 15)
		Building trust with communities [28]	‘*Members of our community know they can go and ask [their pharmacist Rabbi] anything… he is a trusted and respected figure in our community… it is important to us to know we can trust him’*. (Participant 11)
			‘*Having someone they could confide in by speaking their own language and removing that stress of not knowing how to say, how to communicate’*. (Participant 6)
			‘*I said to (pharmacist) “for Ramadan” and she knew exactly what my worry was because she was a Muslim too’*. (Participant 1)
			‘*I could go back in and ask any question about my medicines if (the member of staff) was working thereand discussed how they would ask themif they can explain me in my language and then I feel comfortable to ask anything’*. (Participant 7)
		Cultural considerations for home medicines reviews with aboriginal patients [56]	‘*The health service people are people you trust, people that look after you, people you know. If they organise it then it must be okay. Also they know about our family, where to find us and can organise transport and the right time’*.
			‘*[If it was organised by their Aboriginal Health Service] then youcan trust that the pharmacist is appropriate and that it [HMR] is for your benefit’*.
			‘*The health worker breaks things down for us, so that we can understand’*.
			‘*[If the Aboriginal Health Worker was a community member] I don't want her to know my business’*.
		Linguistic sanguinity and multilingual skill desirability [55]	‘*I have never been to doctors and pharmacists who are Westerners, in that clinic [i.e. the one visited by the participant] all the doctors and staff [have] the same language and cultural background [so] they are able to understand us’*. (Participant 27)
		Influence of researcher's ethnic background on education program [61]	‘*You [the researcher] have lived in an Arabic country and share the same background as ours, so that's why you understand our conditions and circumstances’*. (Focus group 6)
		Communication with healthcare providers [54]	‘*I always asked for and travelled to see my doctor as he speaks Arabic’*. (Focus group 1)
		Language and cultural barriers [58]	‘*It is far easier when it is explained in your own language. For any person they will always prefer their own language, their own community and have someone that can translate and understand things’*. (Respondent 2)
	A training necessity: Cultural competence	Cultural competence education for pharmacists [62]	‘*I think the non‐Black pharmacists, if they don't actually go through this already, could benefit from some … anti‐oppression or kind of cultural awareness or even just the basic intro to psychology type training stuff’*.
		Suggestions for improvement [63]	‘*They should learn customer service to deal with people that do not understand [the English language] or are confused’*. (Participant 2)
		Acknowledging cultural beliefs and recognising potential stigma [24]	‘*Some kind of training needs to be given around this’*. (Participant 18)
			‘*Have more culturally sensitive [underpinnings]’*. (Participant 5)
		Providing medicines advice tailored to diverse patient cohorts [28]	‘*More learning about the people's culture who live in the area around (the pharmacy)’*. (Participant 6)
			‘*Speaking to me, speaking to my neighbours, asking us Muslims because we can tell to them if it is something they do not know about’*. (Participant 2)
			‘*Training for (pharmacy teams) around other cultures, or cultures different to their own so they can be more aware’*. (Participant 5)
		Cultural considerations for home medicines reviews with aboriginal patients [56]	‘*It can't just be anyone. They have to culturally appropriate or they could offend someone’*.

Abbreviations: GP = general practitioner (medic); HMR = home medication review; NZ = New Zealand.

**Table 6:** Theme 4: Addressing language and communication.

**Overarching third‐order construct (termed ‘theme’) developed by the research team**	**Third‐order subtheme developed by the research team**	**Second‐order constructs (interpretations made by the original study authors)**	**First‐order construct (quotations taken directly from participants of the original studies)**
Supporting with possible language and communication barriers	Verbal and non‐verbal forms of communication	Obtaining and understanding health information [63]	‘*I never called. I like to do it more in person because the communication is hard for me’*. (Participant 23)
			‘*I have never called a pharmacist to ask questions because they do not speak Spanish and I do not speak English’*. (Participant 22)
		Language barriers [63]	‘*The pharmacist speaks Spanish where I go to regularly, but the pharmacist at night time they don't’*. (Participant 13)
		Experiences with a pharmacist or pharmacy staff [63]	‘*The only problem that I have found is that they do not speak Spanish. Sometimes I feel afraid to talk to them because of that’*. (Participant 21)
		Suggestions for improvement [63]	‘*More empathy with Spanish speakers. For example, do not say ‘I am sorry I don't have anybody’ [to interpret] when it's about health’*. (Participant 12)
			‘*I think they should learn languages because this is a diverse country and they should learn how to handle people from different backgrounds’*. (Participant 20)
		Language as a barrier [58]	‘*They went to see Chinese doctors; they were NZ registered practitioners that spoke Chinese. … The doctor told them where they could get there medicines and how many times a day to take the medicine’*. (Respondent 9)
		Communication barriers [53]	‘*Here we are second language and then I can't explain everything for the doctors for example, do you know what I mean? Very hard for us, honestly, everybody from Somalia’*. (Focus group 1)
			‘*…Usually you get asked ‐ ah ‐ Did the doctor explain how to take it? Sometimes they explain sometimes, but not all the time. And of course if they explain, but then if you don't speak the language…’*
		Addressing language and communication barriers (Robinson et al. [24])	‘*If someone is not a native English speaker and suddenly being expected to take all of this medication and all this jargon of names… as well as being expected to know what (the healthcare professional) mean when they give out advice’*. (Participant 10)
			‘*People who speak languages that are spoken only, where they cannot be written down’*. (Participant 17)
			‘*Translating all of the discussion back to the patient's first language so they're involved… so they feel involved and listened to’*. (Participant 19)
		Face‐to‐face connections and reassurance (Robinson et al. [28])	‘*I need to watch people's mouths move… it's easier to understand that way if English is not your first language like me’*. (Participant 12)
			‘*Vital that a person understands what (medications) they're taking and why… they can't even speak English so how do you best get that message about medicines across to them? ’* (Participant 5)
		Language barrier [57]	‘*We just show [the pharmacist] the prescriptions and take what they give us. It's impossible for us to ask why we should take this medication and what effect it has’*.
		Language and cultural barriers [58]	‘*Having the label in Chinese would make it a lot easier for many migrants that are Chinese’*. (Respondent 8)
		Therapeutic needs [54]	‘*I would love to see consumer medicines information in Arabic added to the English Version in place. It would help knowing the recommended doses, possible side effects, and contraindications of each medicine’*. (Focus group 1)
		Understanding medicine‐related instructions [55]	‘*It should be more simpler English, for example it says take 2 tablets a day, then in the bracket should be 1 in the morning and 1 in the afternoon … because it is a bit confusing when it says 2 a day, I could take maybe 2 together’*. (Participant 22)
			‘*It would be very helpful, if a Chinese sticker could be put on top of the English package but actually that's what my pharmacist did for me anyway because at the beginning we didn't understand so the pharmacist wrote down all the information in Chinese on a piece of paper on the package; for example before meal, after meal, [this made] everything clear’*. (Participant 27)
		Usefulness of available health information [55]	‘*Because the chart has pictures and has both Chinese and English, that's how I communicate with them’*. (Participant 26)
			‘ *… It would be convenient. I prefer Chinese and English version [of medicine information] come together’*. (Participant 24)
		Differentiation between meds [55]	‘*They [the brand names] all have English names and all start with different kind of letters, so I will remember H for that … But I never learnt English it is just the letter [i.e. the shape of the letter]’*. (Participant 28)
			‘*They change the carton sometimes, but the name is the same. No I don't rely on the box because it changes’*. (Participant 3)
			‘*Sometimes color and labeling are totally different‐ looks like it's not the same tablet (when in fact it is)’*. (Participant 23)
			‘*I try to recognize the package … That's the reason why I absolutely hate it when they get me to change brands’*. (Participant 30)
		Addressing language and communication barriers [24]	‘“*Why can't they put things on the labels in Urdu for me?” so it can be easier for reading it’*. (Participant 15)
		Face‐to‐face connections and reassurance [24]	‘*Sometimes people not say anything, their face tells you. It is harder to read someone when you not in the place with them… I think it's the facial expressions, maybe the reactions, maybe the body language’*. (Participant 3, via an interpreter)
			‘*It's not the same when you talk on the phone… (the pharmacist) want to discuss with you what is the medicine, why you are on the medicine, is it helpful? But sometimes I need to point to the box, want to describe my answer like that, or want to point to the things, but I cannot if it is talking on the phone… like my legs when I have the swelling in my ankles’*. (Participant 1)
		Verbal and non‐verbal communication [28]	‘*Including the specific medicines' details on the label or something, like when in the day you take it or if you can have it with a cuppa’*. (Participant 5)
			‘*Should be written in English… otherwise there becomes no incentive to learn English if it is always for you in Punjabi’*. (Participant 7)
		Adapting home medicines reviews to aboriginal patients' needs [56]	‘*It would have been good in the pharmacist had left some written information, simple to understand, to show my family and read later’*.
	Using interpreters: Formally or informally	Communication barriers [58]	‘*…If my mum ask for Swahili they bring Congolese who can speak Swahili which is totally different to the Swahili we know because they mix with French, and my mum doesn't speak any French at all’*. (Focus group 4)
		Communication with healthcare providers [54]	‘*I always go with my daughter to see my pharmacist. She helps me understand his recommendations as I do not understand English very well’*. (Focus group 1)
		Social networks [55]	‘*If I had any questions I would just ask [my] daughter because my husband and I don't really speak English so we can't communicate [with others]’*. (Participant 27)
		Raising awareness through community‐centred support [24]	‘*I am member of several WhatsApp® groups (where)… people who need the help, they explaining the problem in Arabic and me and my friends translate to the right words so they can explain to pharmacist’*. (Participant 15)
		Addressing language and communication barriers [24]	‘*I know they would not have gone (to seek medication advice) if they did not have me for the translating… I know that the people in the Arabic community will leave to suffer in silence rather than speaking up… it is why translating the language gap is so important’*. (Participant 15)
		Language barriers [63]	‘*My English is pretty bad. It's easier for me to talk and describe what I need when they have people who speak Spanish’*. (Participant 23)
			‘*It would improve my pharmacy experience if they have interpreters at night’*. (Participant 22)
			‘*The time to wait for a translator is long’*. (Participant 9)
			‘*When I talk to them and they speak to me in English I just say yes, yes. But when they speak to me in Spanish or when they use a translator I ask questions’*. (Participant 13)
			‘*Interpreters are helpful when they are there’*. (Participant 6)
		Verbal and non‐verbal communication [28]	‘*How the interpreter can be with the GP for his appointment, but why not with pharmacist for his?’* (Participant 8)
			‘*When I need translator for GP appointment, I always get told to have appointment the next day so they can organise someone to speak Punjabi with me. But (in the pharmacy), they cannot do this as it is more walk‐in not normally booked day or time… maybe it is possible to do the same?’* (Participant 9)

Abbreviations: GP = general practitioner (medic); NZ = New Zealand.

### 3.5 Theme 1: Navigating Pharmacy Systems Across the Globe

This theme (Table 3) explored the challenges experienced by people from ethnic minority communities when accessing pharmacy systems and medicine services, particularly considering challenges associated understanding the health system in the place they are living. This theme comprised of three subthemes focusing on (i) understanding, and raising awareness of, pharmacy services; (ii) comparing access to medication in different countries; and (iii) cost implications associated with taking medicines.

#### 3.5.1 Understanding, and Raising Awareness of, Pharmacy Services

The majority of the included studies reported participants not knowing what pharmacy services were available to them in the country they currently lived in, compared to their previous home country; this was of particular relevance for people with refugee status and those seeking asylum. Examples of pharmacy services and support that participants did not know were available included medication reimbursement systems [58, 61] and medicines review services [24, 28, 56]. Offering simple explanations about over the counter medicine availability, and managing expectations around the supply of medicines with or without a prescription, was deemed essential to support gaps in understanding [53, 58].

I got this feeling a lot of people doesn't really know what service… the pharmacy do offer for medicines … He or she maybe doesn't know there is such a thing as a medicine review.

(Robinson et al. [24])

Participants recognised the need to better raise awareness of the services available from pharmacies, and the medicines support and information on offer from the pharmacist or wider members of the team [24, 28, 61]. Suggestions for how to do this included flyers in places of worship or areas specific to ethnic minority communities, such as local Halal food shops, use of community leaders to spread awareness, and adverts on local radio stations [24, 28].

Hearing about it from people they trust… like the Imam, or equivalent if they aren't Muslim, that's probably going to be someone in their community they listen to and respect.

(Robinson et al. [28])

#### 3.5.2 Comparing Access to Medication in Different Countries

Across three studies, participants discussed the differences between access to medication in their home country compared to their new one [53, 58, 63]; one example of this included antibiotics that could be purchased over‐the‐counter without a prescription [53]. In these instances, feelings of frustration were associated with challenges in understanding and navigating the new healthcare system, as well as misconceptions around medicine suitability. One participant described not realising the risks of taking purchasing medications without a prescription when it was so readily available.

Even some of the strong medicines here, like antibiotics, we don't call it strong back home because you buy it just from anyone at a shop without any script.

(Bellamy et al. [53])

#### 3.5.3 Cost Implications Associated With Taking Medicines

Pertinent to individuals who had migrated to a new country, medication costs were reported to influence a person's ability to take medicines. Participants described instances where they had not been able to afford their medication and, as a result, missed treatment for a period of time [58, 61]. Examples provided also related to increased costs of healthcare and insurance dependent on an individual's residency and visa status in a particular country [58]. Similar examples were discussed in relation to non‐prescription medication, with participants reporting challenges with affording treatments for common ailments and illnesses; the impact of this on their mental health and wellbeing was also shared, with people describing feelings of insecurity [58, 61].

Some people they say, ‘We cannot afford these’ and so they do not have them [medicines].

(Babar et al. [58])

### 3.6 Theme 2: Understanding Cultural Needs and Beliefs That May Influence Medicines Use

Culturally centred pharmacy services should be cognisant of possible cultural and religious preferences when aiming to be equitable and inclusive. This theme (Table 4) aimed to acknowledge the cultural needs and beliefs that have been evidenced to influence medicine use amongst individuals from ethnic minority backgrounds. In particular, appreciating (i) cultural and religious preferences regarding medicines; and (ii) the use of traditional medicines as alternative therapies.

#### 3.6.1 Appreciating Cultural and Religious Preferences Regarding Medicines

A number of examples were provided across the included studies to demonstrate the need to underpin service design and delivery with cultural competence and knowledge of possible cultural or religious preferences. These included knowledge of restricted excipients that may affect a person taking their medications, for instance, alcohol or gelatine [24, 28, 58]; understanding whether consultations should be undertaken by a healthcare professional of the same sex to encourage access to, and engagement with, services [58]; and appreciating the impact of religious practices, such as fasting and pilgrimage, and how these may affect medication adherence or require tailored medicines advice [24, 28].

My own preferences… these should be the things pharmacists and doctors consider for us (Arabic community) but maybe they don't know them, maybe they aren't taught them, but there's a simple way of asking me to find this stuff out.

(Robinson et al. [28])

#### 3.6.2 Use of Traditional Medications

Several examples of understanding cultural norms and behaviours that centred around traditional medicines use were shared within the included studies. Participants reported familiarity and reassurance when using traditional medicines, perceiving they understood them more and that they were safer than Western medications, especially for minor ailments [24, 58, 63]. Participants discussed only resorting to Western medications if it was a serious illness or if the traditional herbal medicine had not worked [24, 63]. Some participants expressed the need for healthcare professionals to respect this and continue to treat them without judgement of their traditional practices [24].

We know what is best. We know it doesn't have side‐effects [talking about natural/traditional remedies] … It doesn't harm anything so we just do it.

(Babar et al. [58])

### 3.7 Theme 3: Strengthening Relationships With Pharmacists and Pharmacy Teams

This theme (Table 5) synthesised factors that strengthened relationships with pharmacists and other healthcare professionals, and reflected how these impacted or influenced the experiences of those from ethnically minoritised communities. Specifically, this concerned (i) lived‐experiences of judgement and stigma, versus ways to change the narrative; (ii) connections between culture and community; and (iii) necessary cultural competence training requirements for staff.

#### 3.7.1 Judgement and Stigma Versus Ways to Change the Narrative

The majority of studies shared examples of ethnicity‐based judgement and stigmatisation that people have faced from healthcare professionals, which impacted their care experiences [24, 56, 57, 60, 61, 62, 63]. Some participants described limited interaction with healthcare professionals on the basis of the language they spoke [57, 61, 63], whereas others felt that healthcare professionals held preconceived judgements about their social standing, based on their ethnicity [60, 62]. In some instances, participants expressed a lack of trust for Western healthcare professionals, instead, preferring to follow the advice of a doctor from their home country [61].

Knowing how to remove what is the perceived power balance between me as the Māori that goes in, versus that very flash looking Pākehā [NZ European] behind the bench that I think is looking down on me.

(Hikaka et al. [60])

Conversely, several studies discussed the importance of pharmacists playing a key role in changing that narrative – specifically, by facilitating positive relationships with people from ethnic minority communities in order to inspire confidence and improve rapport [28, 59, 60, 62]. Examples included building credibility amongst communities to create an open dialogue and safe space, where people could gain confidence and speak freely [28].

#### 3.7.2 Shared Experiences of Culture and Community

Patients and healthcare professionals who shared the same cultural background was viewed as a factor that enabled culturally centred pharmacy services to be delivered. Reported advantages of this were attributed to establishing a deeper working relationship between patient and professional, supporting feelings of safety and reassurance when receiving care, and enabled people to feel respected and heard [24, 28, 54, 55, 56, 58, 59, 61, 62]. One participant from a South Asian community expressed relief when their pharmacist understood concerns about Ramadan and medication adherence, due to them both following the Muslim faith [28]. However, some concerns were raised if the person required treatment for a health condition that they perceived to carry a stigma or be embarrassing – where having a healthcare professional from their community could be viewed as uncomfortable [24, 56].

People will not discuss it (mental health conditions) … especially if the pharmacist was a member of the community himself… that way it might become common knowledge and affect the family's reputation.

(Robinson et al. [24])

#### 3.7.3 Necessary Cultural Competence Training

One reported approach to facilitate more positive relationships between patient and pharmacist, and improve cultural understanding, was for pharmacy staff to undergo cultural competency training [24, 28, 56, 62, 63]. Learning about other cultures and how to accommodate them was deemed important for building positive relationships and improving care. This was viewed as an essential strategy for all members of the pharmacy team, not solely the pharmacist, recognising the wider role of counter assistants and pharmacy technicians in providing culturally‐centred medicines advice and support [24, 28, 56, 62, 63].

Training for (pharmacy teams) around other cultures, or cultures different to their own, so they can be more aware.

(Robinson et al. [28])

### 3.8 Theme 4: Addressing Possible Language and Communication Barriers

This theme (Table 6) captured the issues surrounding language that act as a barrier to communication between healthcare professionals and people from ethnic minority communities. These include (i) verbal and non‐verbal forms of communication, as well as (ii) the formal use of interpreter services compared with the informal role that family and friends may play in aiding communication.

#### 3.8.1 Verbal and Non‐Verbal Forms of Communication

Verbal communication was widely discussed in 11 of the 13 included studies [24, 53, 54, 55, 57, 58, 59, 60, 61, 63]. Spoken health communication was described as invaluable when done correctly, however, many barriers were recognised that could impact delivery of culturally centred pharmacy services. Several participants, who were non‐native English speakers, described not asking pharmacists for advice as they did not speak the same language [55, 63]. Others discussed the challenges of trying to understand not only an additional language, but medical jargon on top of this [24, 28, 53], with requests for issues relating to medication to be explained using simpler terms or in basic English [55]. Additional challenges were acknowledged for those who communicated in solely spoken languages, as this limited the options for overcoming language barriers through use of alternative written communication [24]. There was an overall desire for healthcare professionals to translate information for people from ethnic minority backgrounds to support a better understanding of health and safe medicine use [24, 54, 58, 61, 63].

We just show [the pharmacist] the prescriptions and take what they give us. It's impossible for us to ask why we should take this medication and what effect it has.

(White et al. [57])

Alternative means of communication were widely discussed as being an enabler to support safe and effective medicine use, despite language barriers. Such examples included translating medication labels and information leaflets into languages other than English [24, 28, 54, 55, 58], using pictures or illustrations (such as a sun or a moon) to indicate the timings of medication doses [55], and the provision of translated written information that patients could retain [28, 56]. Several participants described a reliance on recognising the packaging or colour of tablets to distinguish between their different medications as they could not understand English; thus, concerns were raised when dispensed medication came from an alternative manufacturer [55].

I would love to see consumer medicines information in Arabic added to the English version… it would help knowing the recommended doses, possible side effects.

(Focus group 1, el Hait et al. [54])

#### 3.8.2 Using Interpreters: Formally and Informally

Given the emphasis placed on language translation as a means of overcoming a number of communication challenges for people from ethnic minority communities, the use of interpreter services was developed into its own distinct sub‐theme [28, 55, 58, 63]. Examples were shared across the studies noting the differences in how a person may reach out for interpretation support, with some participants using informal contacts (such as family members or close friends) and others using the interpreters provided more formally through healthcare services [28, 55, 58, 63]. In one study, participants shared experiences of supporting community centred translation, digitally, within a WhatsApp group [24].

Although it was recognised that interpreters can be helpful when they are available [63], participants did raise concerns around ensuring the quality that should underpin such services if they are to be provided more formally. It was deemed equally important to ensure that the correct dialect, as well as the correct language, needs were met by the interpreter services [58]. There were also challenges noted that related to second language proficiency levels, and limited cross‐sector availability, of interpreters; participants noted that it was common for wait times to be long before seeing an interpreter [63], which not only meant that appointments were delayed, but also meant that it was a less than ideal situation for those experiencing acute illness that required time‐critical care from a pharmacist. Participants described it being much more difficult to get an interpreter in the pharmacy compared to other healthcare settings, like general practice, due to the walk‐in nature of community pharmacy without requiring a pre‐booked appointment [28].

How the interpreter can be with the GP for his appointment, but why not with pharmacist for his?.

(Robinson et al. [28])

### 3.9 Recommendations for Implementing and Delivering Culturally‐Centred Pharmacy Services

**Figure 3:**
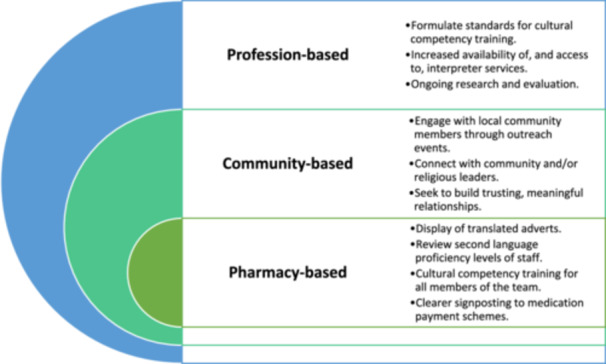
Recommendations for implementing and delivering culturally centred pharmacy services: an individual pharmacy‐based, community‐based, and systems‐based approach.

The findings of this review have wide‐ranging implications for practising pharmacists, as well as health policymakers and those involved in the initial education and training of pharmacy teams. Figure 3 illustrates a template of recommendations for key stakeholders as a way to support the implementation and delivery of culturally centred pharmacy services tailored for ethnically minoritised populations – it takes into consideration, and offers suggestions on, how to achieve such services on: (i) an individual pharmacy‐, (ii) community‐ and (iii) profession‐based level. Such recommendations are multidimensional, echoing a micro‐meso‐macro framework approach [64, 65], and are intended to encompass points that span across the themes from this meta‐ethnographic synthesis, thus appreciating that efforts should adopt a multifaceted approach to improving culturally centred pharmacy services. Crucially, ongoing research and evaluation is required to evaluate such approaches; whilst service acceptability is an important consideration, future research should also seek to measure and review service effectiveness, working alongside those with lived‐experience towards achieving equitable outcomes for people from ethnically minoritised groups.

## 4 Discussion

This is the first meta‐ethnographic systematic review of the global literature to synthesise and examine approaches used in the provision of culturally centred pharmacy services tailored to people from ethnic minority communities. Four overarching themes (third‐order constructs) were developed which centred around: experiences of navigating pharmacy health systems; understanding cultural needs and beliefs that may influence medicines use; strengthening relationships with pharmacists and pharmacy teams; and appreciating strategies to tackle such inequalities, namely, through cultural competence training and addressing language and communication barriers.

The profession of pharmacy has demonstrated recent commitment to addressing ethnic inequity, particularly in relation to service design and delivery to best meet the needs of culturally diverse, marginalised patient populations [23, 31, 66]. Work has been undertaken that inwardly examines the training of pharmacy professionals and members of the entire pharmacy team, with specific emphasis placed on cultural competence training [18] and interrogating the education and training of pharmacy students [19, 67, 68]. However, even when pharmacists described having access to such cultural training, or possessing this knowledge, there have been instances of inertia in taking action due to fears of low confidence [69, 70], overstepping [71] or unintentionally using the incorrect language or terminology [72, 73]. Future research could seek to outwardly examine approaches used across other healthcare professional groups in a bid to further learning and share best practice across the entire multi‐disciplinary team. Similar findings have been reported by colleagues in dentistry, with efforts taken to address ethnic inequity in oral health care across the wider profession by first placing educational focus on cultural competence for dental students [74, 75, 76]. This also echoes work done within medicine [77, 78, 79] and nursing [80, 81]. While many efforts across the health and social care professional groups have centred on the initial education and training of future members of the profession, there still remains a gap in knowledge about the *delivery* of services. Next, inclusive co‐design and co‐production methodologies should seek to bring together the voices of community members, alongside clinicians and students, to suggest recommendations to implement this learning to better deliver equitable and accessible pharmacy services [28].

The provision of understandable medicines advice is especially pertinent for those from ethnically minoritised groups who may speak another language from the place they are residing [23, 39, 40, 71, 82, 83, 84, 85, 86]. Although professional interpreter services have previously facilitated healthcare consultations in outpatient clinical settings with success [87, 88, 89, 90], there have been historical challenges around access to such services within community pharmacy [39, 91, 92]. When considering how best to integrate interpreters to support culturally competent medicines consultations, future qualitative studies could explore access to, and experiences of, such services from the perspectives of patients and members of the pharmacy team.

There is an unfulfilled need regarding the provision of multilingual medication labels reported in the literature [93, 94, 95], despite evidence that patient comfort and satisfaction with healthcare services increased with the use of translated materials [96]. Whilst computer‐based translational resources have been perceived as easily accessible and helpful with supporting health literacy within American community pharmacy, these were infrequently used [93, 94, 97]. ‘The Written Medicine’, a UK web‐based software, has been developed to tackle this issue through bilingual prescription labels which include English alongside the person's native language [98]. Future research should explore the uptake and utilisation of such software within community pharmacies, as well as the impact it could have in supporting health literacy and culturally centred medicines taking. By recognising possible cultural influences that can affect medicines taking, efforts should be made to further support health literacy for those from ethnic minority communities that may benefit [53, 55, 57, 58, 61]. Wider studies within health and social care research have reported some evidence of lower levels of health literacy amongst some minority groups, including individuals from ethnically minoritised communities [40, 85, 99, 100]; this has been attributed to possible misunderstandings between healthcare professionals and patients, which may affect medication non‐adherence and result in poorer health outcomes.

Whilst the approach to this research was novel, it is recognised that there were some limitations; firstly, as the process of conducting meta‐ethnographic reviews is primarily interpretive, the results may not always be transferable to all cultures or all lived‐experiences of people from ethnic minority populations when accessing pharmacy services. Researchers were not able to tease apart the differences of views of different cultures to draw specific or more nuanced conclusions and recommendations. Future research may seek to work collaboratively with members of minoritised communities to further explore, co‐design and co‐evaluate culturally centred pharmacy services. Furthermore, the studies were restricted to adult cohorts and to those published in the English language only; these restrictions may have limited the populations studied and therefore may not have captured views of wider marginalisation. The meta‐ethnography was developed and conducted from a pharmacy‐centred angle, and similarities may have been found if the study had been broadened to wider healthcare settings. However, the decision was made to focus this review solely on pharmacy services due to their key accessibility, particularly in areas of high deprivation, where they are often the first port of call for healthcare advice [30]. There was diversity included within the studies in this review; this work synthesised the current global literature base, across a range of countries. Whilst distinctive cultural traits were not explored, the authors do recognise this may have potential to influence perceptions and, consequently, how people may access healthcare. Comparably, there was no emphasis on the role that immigration status, ethnicity, gender or religion played in obtaining health care; the research team recognise that members of these groups have additional challenges that may require further, independent investigation [101].

## 5 Conclusion

This novel meta‐ethnographic systematic review synthesised the findings from 13 international studies to examine approaches used in the provision of culturally centred pharmacy services for people from ethnic minority communities. From this synthesis, a template of recommendations has been developed to further implement and deliver such services on an individual pharmacy‐, community‐ and profession‐basis. When seeking to improve the tailoring of pharmacy services for ethnically minoritised groups, pharmacists and policymakers should seek to: raise awareness of pharmacy services, build stronger relationships with minority communities, and facilitate access to interpretation services. Consideration should also be given to further exploring and analysing the provision of cultural competence training to members of the profession, in a bid to supporting equitable care consultations throughout a person's career in pharmacy. Future research should collaboratively involve people with lived‐experience alongside practicing pharmacists, pharmacy team members, wider members of the health and care multi‐disciplinary team, and policymakers to further co‐design and evaluate culturally centred pharmacy services.

## Author Contributions

**Caitlin Shaw:** data curation, investigation, writing–review and editing, project administration, formal analysis. **Ghalib Khan:** supervision, resources, writing–review and editing, validation. **Thorrun Govind:** supervision, resources, writing–review and editing, validation. **Anna Robinson‐Barella:** conceptualisation, methodology, writing–original draft, writing–review and editing, supervision, visualisation.

## Ethical Statements

There was no need to undergo or seek ethical approval for this work, given it is a systematic review.

## Consent

The authors have nothing to report.

## Conflicts of Interest

The authors declare no conflicts of interest.

## Supporting information

Supporting information.

## Data Availability

The data that support the findings of this study are available from the corresponding author upon reasonable request.
