# Supplementary material for: Implementing and Delivering Culturally Centred Pharmacy Services Tailored to Ethnically Minoritised Populations: A Qualitative Systematic Review and Meta‐Ethnography
Source: Health Expect. 2025 Jan 31;28(1):e70165. doi: 10.1111/hex.70165 (PMC11783237; doi:10.1111/hex.70165)
Supplement: Supplementary file 1 — Supporting information. [file HEX-28-e70165-s001.docx]

**Implementing and delivering culturally-centred pharmacy services tailored to ethnically minoritised populations: a qualitative systematic review and meta-ethnography.**

**SUPPLEMENTARY FILE**

Items:

Appendix A: PRISMA 2020 checklist

Appendix B: Database search strategy

Appendix C: Study quality appraisal

Appendix A: PRISMA Checklist

Table 1: PRISMA Checklist

| **Section and Topic** | **Item #** | **Checklist item** | **Location where item is reported** |
| --- | --- | --- | --- |
| **TITLE** | | |  |
| Title | 1 | Identify the report as a systematic review. | Title, abstract, introduction |
| **ABSTRACT** | | |  |
| Abstract | 2 | See the PRISMA 2020 for Abstracts checklist. | 1-2 |
| **INTRODUCTION** | | |  |
| Rationale | 3 | Describe the rationale for the review in the context of existing knowledge. | 3-4 |
| Objectives | 4 | Provide an explicit statement of the objective(s) or question(s) the review addresses. | 4 |
| **METHODS** | | |  |
| Eligibility criteria | 5 | Specify the inclusion and exclusion criteria for the review and how studies were grouped for the syntheses. | 4-7 |
| Information sources | 6 | Specify all databases, registers, websites, organisations, reference lists and other sources searched or consulted to identify studies. Specify the date when each source was last searched or consulted. | 5-6 |
| Search strategy | 7 | Present the full search strategies for all databases, registers and websites, including any filters and limits used. | 5-6, Tables 2-5 in Supp. file |
| Selection process | 8 | Specify the methods used to decide whether a study met the inclusion criteria of the review, including how many reviewers screened each record and each report retrieved, whether they worked independently, and if applicable, details of automation tools used in the process. | 4-6 |
| Data collection process | 9 | Specify the methods used to collect data from reports, including how many reviewers collected data from each report, whether they worked independently, any processes for obtaining or confirming data from study investigators, and if applicable, details of automation tools used in the process. | 5-6 |
| Data items | 10a | List and define all outcomes for which data were sought. Specify whether all results that were compatible with each outcome domain in each study were sought (e.g. for all measures, time points, analyses), and if not, the methods used to decide which results to collect. | N/A |
|  | 10b | List and define all other variables for which data were sought (e.g. participant and intervention characteristics, funding sources). Describe any assumptions made about any missing or unclear information. | N/A |
| Study risk of bias assessment | 11 | Specify the methods used to assess risk of bias in the included studies, including details of the tool(s) used, how many reviewers assessed each study and whether they worked independently, and if applicable, details of automation tools used in the process. | 6 |
| Effect measures | 12 | Specify for each outcome the effect measure(s) (e.g. risk ratio, mean difference) used in the synthesis or presentation of results. | N/A |
| Synthesis methods | 13a | Describe the processes used to decide which studies were eligible for each synthesis (e.g. tabulating the study intervention characteristics and comparing against the planned groups for each synthesis (item #5)). | N/A |
|  | 13b | Describe any methods required to prepare the data for presentation or synthesis, such as handling of missing summary statistics, or data conversions. | N/A |
|  | 13c | Describe any methods used to tabulate or visually display results of individual studies and syntheses. | 7, 10, Tables 2-6 |
|  | 13d | Describe any methods used to synthesize results and provide a rationale for the choice(s). If meta-analysis was performed, describe the model(s), method(s) to identify the presence and extent of statistical heterogeneity, and software package(s) used. | N/A |
|  | 13e | Describe any methods used to explore possible causes of heterogeneity among study results (e.g. subgroup analysis, meta-regression). | N/A |
|  | 13f | Describe any sensitivity analyses conducted to assess robustness of the synthesized results. | N/A |
| Reporting bias assessment | 14 | Describe any methods used to assess risk of bias due to missing results in a synthesis (arising from reporting biases). | N/A |
| Certainty assessment | 15 | Describe any methods used to assess certainty (or confidence) in the body of evidence for an outcome. | N/A |
| **RESULTS** | | |  |
| Study selection | 16a | Describe the results of the search and selection process, from the number of records identified in the search to the number of studies included in the review, ideally using a flow diagram. | 7-8 |
|  | 16b | Cite studies that might appear to meet the inclusion criteria, but which were excluded, and explain why they were excluded. | 8, Figure 1 |
| Study characteristics | 17 | Cite each included study and present its characteristics. | 9, Table 2 |
| Risk of bias in studies | 18 | Present assessments of risk of bias for each included study. | 9, Supp. File |
| Results of individual studies | 19 | For all outcomes, present, for each study: (a) summary statistics for each group (where appropriate) and (b) an effect estimate and its precision (e.g. confidence/credible interval), ideally using structured tables or plots. | N/A |
| Results of syntheses | 20a | For each synthesis, briefly summarise the characteristics and risk of bias among contributing studies. | N/A |
|  | 20b | Present results of all statistical syntheses conducted. If meta-analysis was done, present for each the summary estimate and its precision (e.g. confidence/credible interval) and measures of statistical heterogeneity. If comparing groups, describe the direction of the effect. | N/A |
|  | 20c | Present results of all investigations of possible causes of heterogeneity among study results. | N/A |
|  | 20d | Present results of all sensitivity analyses conducted to assess the robustness of the synthesized results. | N/A |
| Reporting biases | 21 | Present assessments of risk of bias due to missing results (arising from reporting biases) for each synthesis assessed. | 9, Supp File |
| Certainty of evidence | 22 | Present assessments of certainty (or confidence) in the body of evidence for each outcome assessed. | N/A |
| **DISCUSSION** | | |  |
| Discussion | 23a | Provide a general interpretation of the results in the context of other evidence. | 22-24 |
|  | 23b | Discuss any limitations of the evidence included in the review. | 22-24 |
|  | 23c | Discuss any limitations of the review processes used. | 23-24 |
|  | 23d | Discuss implications of the results for practice, policy, and future research. | 22-24 |
| **OTHER INFORMATION** | | |  |
| Registration and protocol | 24a | Provide registration information for the review, including register name and registration number, or state that the review was not registered. | Not registered |
|  | 24b | Indicate where the review protocol can be accessed, or state that a protocol was not prepared. | N/A |
|  | 24c | Describe and explain any amendments to information provided at registration or in the protocol. | N/A |
| Support | 25 | Describe sources of financial or non-financial support for the review, and the role of the funders or sponsors in the review. | Title page, 25 |
| Competing interests | 26 | Declare any competing interests of review authors. | Title page, 25 |
| Availability of data, code and other materials | 27 | Report which of the following are publicly available and where they can be found: template data collection forms; data extracted from included studies; data used for all analyses; analytic code; any other materials used in the review. | Appendices in supp. file |

Appendix B: Database search terms

*Full search strategy for all journals = [all terms in row 1 linked using ‘OR] AND [all terms in row 2 linked using ‘OR’] AND [all terms in row 3 linked using ‘OR’] AND [all terms in row 4 linked using ‘OR’]*

Table 2: Search Strategy used in MEDLINE

| Cultural Competency/ or Culturally Competent Care/ or cultural diversity/ or diversity, equity, inclusion/ or cross-cultural comparison/ or (cultur* adj3 (competen* or appropriate* or sensitiv* or aware* or safe* or tailor* or develop* or factor* or specific* or differen* or need* or cent?red or humility or understanding)).mp. or "cross-cultural care".mp. or "cross-cultur*".mp. or "trans-cultur*".mp. |
| --- |
| Education/ or Pharmacy/ or Pharmacies/ or Pharmacists/ or Pharmaceutical Services/ or community pharmacy services/ or drug information services/ or medication therapy management/ or pharmaceutical services, online/ or Pharmacy Service, Hospital/ or prescriptions/ or pharmacy.mp. or pharmacies.mp. or pharmacist*.mp. or pharmac*-led.mp. or pharmac*-facilitated.mp. or pharmac*-managed.mp. or pharmac*-supervised.mp. or ((drug* or medicine* or medication*) adj3 (review or information or monitor* or manage*) adj3 (service* or intervention*)).mp. or ((pharmacy or pharmacist* or pharmaceutical) adj3 (intervention* or service* or manage*)).mp. or ((drug* or medicine* or medication*) adj3 adher*).mp. or "prescription drug monitoring".mp |
| "Ethnic and Racial Minorities"/ or "Emigrants and Immigrants"/ or Undocumented Immigrants/ or "Transients and Migrants"/ or Population Groups/ or African People/ or North African People/ or central african people/ or east african people/ or southern african people/ or west african people/ or sub-saharan african people/ or asian people/ or asian/ or asian/ or central asian people/ or east asian people/ or north asian people/ or southeast asian people/ or west asian people/ or middle eastern people/ or South Asian People/ or black people/ or "black or african american"/ or caribbean people/ or central american people/ or indians, central american/ or "middle eastern and north africans"/ or north american people/ or "american indian or alaska native"/ or indians, north american/ or population groups, us/ or ethnicity/ or "hispanic or latino"/ or mexican americans/ or racial groups/ or "native hawaiian or other pacific islander"/ or oceanians/ or australasian people/ or "australian aboriginal and torres strait islander peoples"/ or pacific island people/ or maori people/ or south american people/ or indians, south american/ or refugees/ or Roma/ or "Asian American Native Hawaiian and Pacific Islander"/ or Indigenous Peoples/ or Arabs/ or Amish/ or Jews/ or BAME.mp. or "Black Asian and Minority Ethnic".mp. or BIPOC.mp. or "Black Indigenous and People of Colo?r".mp. or "ethnic* minorit*".mp. or "ethnic and racial minorit*".mp. or "racial and ethnic minorit*".mp. or co-ethnic*.mp. or "minority ethnic".mp. or "minoriti?ed group*".mp. or "minoriti?ed population*".mp. or "racial minorit*".mp. |
| qualitative.mp or "focus group*".mp. or interview*.mp. or "open-ended".mp. or subjective.mp. or "subjective assessment".mp. |

Table 3: Search strategy used in EMBASE

| transcultural care/ or cultural sensitivity/ or "cross-cultur*".mp. or "trans-cultur*".mp. or (cultur* adj3 (competen* or appropriate* or sensitiv* or aware* or safe* or tailor* or develop* or factor* or specific* or differen* or need* or cent?red or humility or understanding)).mp. |
| --- |
| "pharmacy (shop)"/ or pharmacist/ or community pharmacist/ or hospital pharmacist/ or clinical pharmacist/ or online pharmacy/ or medication therapy management/ or hospital pharmacy/ or pharmacy.mp. or pharmacies.mp. or pharmacist*.mp. or pharmac*-led.mp. or pharmac*-facilitated.mp. or pharmac*-managed.mp. or pharmac*-supervised.mp. or "prescription drug monitoring".mp. or ((drug* or medicine* or medication*) adj3 (review or information or monitor* or manage*) adj3 (service* or intervention*)).mp. or ((drug* or medicine* or medication*) adj3 adher*).mp. or ((pharmacy or pharmacist* or pharmaceutical) adj3 (intervention* or service* or manage*)).mp. |
| ethnic group/ or population group/ or ancestry group/ or ethnicity/ or migrant/ or emigrant/ or immigrant/ or forced migrant/ or refugee/ or asylum seeker/ or black person/ or African American/ or african caribbean/ or african brazilian/ or asian/ or exp East Asian/ or exp central asian/ or exp north asian/ or exp south asian/ or exp Southeast Asian/ or exp West Asian/ or british asian/ or asian american/ or asian continental ancestry group/ or "asian american, native hawaiian and pacific islander"/ or pacific islander/ or oceanic ancestry group/ or Native Hawaiian/ or "Maori (people)"/ or malayo-polynesian people/ or indigenous people/ or american indian/ or alaska native/ or indigenous australian/ or canadian aboriginal/ or hispanic/ or mexican american/ or "romani (people)"/ or BAME.mp. or "Black, Asian and Minority Ethnic".mp. or BIPOC.mp. or multiracial person/ or "ethnic* minorit*".mp. or "racial and ethnic minorit*".mp. or "ethnic and racial minorit*".mp. or co-ethnic*.mp. or "minority ethnic".mp. or "minoriti?ed population*".mp. or Arab/ or "minoriti?ed group*".mp. or Amish/ or Jew/ or African/ or north african/ or African/ or sub-saharan african/ or east african/ or central african/ or southern african/ or west african/ or "Black, Indigenous, and People of Colo?r".mp. or "racial minorit*".mp. |
| qualitative.mp. or interview*.mp. or "open-ended".mp. or subjective.mp. or "subjective assessment*".mp. or "focus group*".mp. |

Table 4: Search strategy used in PsycINFO

| Cultural Competence/ OR cross cultural collaboration/ OR Cross Cultural Differences/ OR cross cultural communication/ OR Diversity Training/ OR cultural diversity/ OR Cultural Sensitivity/ OR Cross Cultural Treatment/ OR (cultur* adj3 (competen* or appropriate* or sensitiv* or aware* or safe* or tailor* or develop* or factor* or specific* or differen* or need* or cent?red or humility or understanding)).mp. OR "trans-cultur*".mp. OR "cross-cultur*".mp. |
| --- |
| Pharmacy/ OR Pharmacists/ OR Community Services/ OR Treatment Compliance/ OR Drug Therapy/ OR pharmacy.mp. OR pharmacies.mp. OR pharmacist*.mp. OR pharmac*-led.mp. OR pharmac*-facilitated.mp. OR pharmac*-supervised.mp. OR pharmac*-managed.mp. OR ((drug* or medicine* or medication*) adj3 (review or information or monitor* or manage*) adj3 (service* or intervention*)).mp. OR ((pharmacy or pharmacist* or pharmaceutical) adj3 (intervention* or service* or manage*)).mp. OR ((drug* or medicine* or medication*) adj3 adher*).mp. OR "prescription drug monitoring".mp. |
| "Racial and Ethnic Groups"/ OR african cultural groups/ OR Asians/ OR chinese cultural groups/ OR japanese cultural groups/ OR Japanese Americans/ OR korean cultural groups/ OR south asian cultural groups/ OR southeast asian cultural groups/ OR vietnamese cultural groups/ OR blacks/ OR caribbean cultural groups/ OR european cultural groups/ OR indigenous populations/ OR alaska natives/ OR american Indians/ OR inuit/ OR pacific islanders/ OR Hawaii natives/ OR "latinos/latinas"/ OR mexican americans/ OR "middle eastern/ and north african cultural groups"/ OR arab/s OR multiracial/ OR "people of color"/ OR romanies/ OR jews/ OR multiculturalism/ OR "racial and ethnic attitudes"/ OR "racial and ethnic differences"/ OR ethnic identity/ OR ethnic diversity/ OR ethnic values/ OR diversity/ OR racial disparities/ OR "race and ethnic discrimination"/ OR racial identity/ OR "racial and ethnic relations"/ OR BAME.mp. OR "Black Asian and Minority Ethnic".mp. OR BIPOC.mp. OR "Black Indigenous and People of Colo?r".mp. OR "ethnic* minorit*".mp. OR "ethnic and racial minorit*".mp. OR "racial and ethnic minorit*".mp. OR co-ethnic*.mp. OR "minority ethnic".mp. OR "minoriti?ed population*".mp. OR "racial minorit*".mp. |
| OR qualitative.mp. OR "focus group*".mp. OR interview*.mp. OR "open-ended".mp. OR subjective.mp. OR "subjective assessment".mp. OR |

Table 5: Search strategy used in CINAHL

| ""cross-cultur*"" OR ""cross-cultural care"" OR ""trans-cultur*"" OR (MH "Leininger's Theory of Culture Care Diversity and Universality") OR ""(cultur* N3 (competen* or appropriate* or sensitiv* or aware* or safe* or tailor* or develop* or factor* or specific* or differen* or need* or cent?red or humility or understanding)" OR (MH "Transcultural Care") OR (MH "Cultural Bias") OR (MH "Cultural Values") OR (MH "Cultural Sensitivity") OR (MH "Cultural Safety") OR (MH "Cultural Diversity") OR (MH "Cultural Competence") |
| --- |
| ""prescription drug monitoring"" OR "((drug* or medicine* or medication*) N3 adher*)" OR "((pharmacy or pharmacist* or pharmaceutical) N3 (intervention* or service* or manage*))" OR "((drug* or medicine* or medication*) N3 (review or information or monitor* or manage*) N3 (service* or intervention*))" OR "pharmac*-managed" OR "pharmac*-supervised" OR "pharmac*-facilitated" OR "pharmac*-led" OR "pharmacist*" OR ""pharmacies"" OR "pharmacy" OR (MH "Education, Pharmacy") OR (MH "Prescription Drug Monitoring Programs") OR (MH "Drug Information Services") OR (MH "Drug Information") OR (MH "Pharmacist Attitudes") OR (MH "Pharmacists") OR (MH "Pharmacy, Retail") OR (MH "Medication Management") OR (MH "Pharmacy Service") |
| (MH "Refugees") OR (MH "Undocumented Immigrants") OR (MH "Immigrants") OR (MH "Transients and Migrants") OR ""minoriti?ed population*"" OR ""minority ethnic"" OR "co-ethnic*" OR ""racial and ethnic minorit*"" OR ""ethnic and racial minorit*"" OR ""ethnic* minorit*"" OR ""Black Indigenous and People of Colo#r"" OR "BIPOC" OR ""Black Asian and Minority Ethnic"" OR "BAME" OR (MH "Minority Groups") OR (MH "South Americans") OR (MH "Roma") OR (MH "Mexican Americans") OR (MH "Hispanic Americans") OR (MH "Amish") OR (MH "Middle Eastern Persons") OR (MH "Kurds") OR (MH "Jews") OR (MH "Pima Persons") OR (MH "Navajo Persons") OR (MH "Native Americans") OR (MH "Inuit") OR (MH "Arctic Peoples") OR (MH "Aboriginal Canadians") OR (MH "First Nations of Canada") OR (MH "Indigenous Peoples") OR (MH "Eastern Europeans") OR (MH "Central Americans") OR (MH "Caribbean Persons") OR (MH "African Americans") OR (MH "Black Persons") OR (MH "Maori") OR (MH "Torres Strait Islanders") OR (MH "Aboriginal Australians") OR (MH "First Nations of Australia") OR (MH "Australasians") OR (MH "Vietnamese") OR (MH "Thais") OR (MH "Laotians") OR (MH "Cambodians") OR (MH "Southeast Asians") OR (MH "South Asians") OR (MH "Pacific Islanders") OR (MH "Hmong") OR (MH "Filipinos") OR (MH "Koreans") OR (MH "Japanese") OR (MH "Chinese") OR (MH "East Asians") OR (MH "Central Asians") OR (MH "Asians") OR (MH "Arabs") OR (MH "Sub-Saharan Africans") OR (MH "Southern Africans") OR (MH "East Africans") OR (MH "Central Africans") OR (MH "West Africans") OR (MH "Africans") OR (MH "Ethnic Groups") OR (MH "Minority Stress") |
| ""open-ended"" OR ""subjective"" OR ""subjective assessment"" OR ""focus group*"" OR "interview*" OR "qualitative" |

Appendix C: Quality appraisal

Table 6: Quality appraisal using the JBI checklist

| **Checklist** | **1** | **2** | **3** | **4** | **5** | **6** | **7** | **8** | **9** | **10** | **Comments** |
| --- | --- | --- | --- | --- | --- | --- | --- | --- | --- | --- | --- |
|  | Is there congruity between the stated philosophical perspective and the research methodology? | Is there congruity between the research methodology and the research question or objectives? | Is there congruity between the research methodology and the methods used to collect data? | Is there congruity between the research methodology and the representation and analysis of data? | Is there congruity between the research methodology and the interpretation of results? | Is there a statement locating the researcher culturally or theoretically? | Is the influence of the researcher on the research, and vice- versa, addressed? | Are participants, and their voices, adequately represented? | Is the research ethical according to current criteria or, for recent studies, and is there evidence of ethical approval by an appropriate body? | Do the conclusions drawn in the research report flow from the analysis, or interpretation, of the data? |  |
| Babar, ZUD *et al.* (2013) (67) | Not applicable | Yes | Yes | Yes | Yes | No | No | Yes | Yes | Yes | Q1: No philosophical perspective stated.  Q1-5: research methodology only stated as “qualitative research”. |
| Bellamy, K *et al.* (2017) (70) | Not applicable | Yes | Yes | Yes | Yes | No | Yes | Yes | Yes | Yes | Q1: no philosophical perspective stated |
| Cantarero-Arévalo, L *et al.* (2014) (75) | Not applicable | Yes | Yes | Yes | Yes | Yes | Yes | Yes | No | Yes | Q1: no philosophical perspective stated  Q9: Stated “this project did not require ethical approval by the Regional Committee on Biomedical Research Ethics.” |
| el Hait, SS *et al.* (2014) (71) | Not applicable | Yes | Yes | Yes | Yes | No | No | No | Yes | Yes | Q1: no philosophical perspective stated  Q8: Not as many quotes from participants in study |
| Gebre, A *et al.* (2023) (76) | Not applicable | Yes | Yes | No | Yes | No | Yes | No | Yes | Yes | Q1: no philosophical perspective stated  Q4: Only one quote was given per subtheme – more should be given if wishing to understand the experience of Black Nova Scotians with community pharmacists as one quote is not indicative of the entire population |
| Hikaka, J *et al.* (2021) (68) | Not applicable | Yes | Yes | Yes | Yes | Yes | Yes | No | Yes | Yes | Q1: This study took a Kaupapa Māori approach for methodology  Q7: The fact this researcher was Māori, the same as the participants, was a key aspect of the intervention and improved outcomes  Q8: More quotes could have been included to represent the participants voices |
| Hikaka, J *et al.* (2023) (69) | Not applicable | Yes | Yes | Yes | Yes | Yes | Yes | Yes | Yes | Yes | Q1: no philosophical perspective stated |
| Knecht, KT *et al.* (2022) (77) | Not applicable | Yes | Yes | Yes | Yes | Uncertain | Yes | Yes | No | Yes | Q1: no philosophical perspective stated |
| Mohammad, A *et al.* (2015) (72) | Not applicable | Yes | Yes | Yes | Yes | No | No | No | Yes | Yes | Q1: no philosophical perspective stated  Q7: Somewhat -- it is addressed that the researcher was not known to participants, and there was no reason for any informal interpreter used to introduce bias, however that is all. |
| Robinson, A *et al.* (2022a) (79) | Not applicable | Yes | Yes | Yes | Yes | No | Yes | Yes | Yes | Yes | Q1: no philosophical perspective stated  Q7: the study states that seven patient champions were employed to help steer the research process and ensure cultural sensitivity throughout |
| Robinson, A *et al.* (2022b) (78) | Not applicable | Yes | Yes | Yes | Yes | Yes | Yes | Yes | Yes | Yes | Q1: no philosophical perspective stated |
| Swain, L *et al.* (2015) (73) | Not applicable | Yes | Yes | Yes | Yes | No | No | Yes | Yes | Yes | Q1: no philosophical perspective stated |
| White, L *et al.* (2012) (74) | Not applicable | Yes | Yes | Yes | Yes | No | No | Yes | Yes | Yes | Q1: no philosophical perspective stated |
